# Abstract Sifter: a comprehensive front-end system to PubMed

**DOI:** 10.12688/f1000research.12865.1

**Published:** 2017-12-21

**Authors:** Nancy Baker, Thomas Knudsen, Antony Williams

**Affiliations:** 1 Leidos, Research Triangle Park, NC, USA; 2 National Center for Computational Toxicology, U.S. Environmental Protection Agency, Research Triangle Park, NC, USA

**Keywords:** PubMed, document retrieval, Microsoft Excel, Visual Basic for Applications, chemistry, toxicology, biology

## Abstract

The Abstract Sifter is a Microsoft Excel based application that enhances existing search capabilities of PubMed. The Abstract Sifter assists researchers to search effectively, triage results, and keep track of articles of interest. The tool implements an innovative “sifter” functionality for relevance ranking, giving the researcher a way to find articles of interest quickly. The tool also gives researchers a view of the literature landscape for a set of entities such as chemicals or genes. The Abstract Sifter is available as a Microsoft Excel macro-enabled workbook application.

## Introduction

Scientists in the biological and medical domains can spend considerable time searching for relevant articles in PubMed, and tools that make the searching more effective will save time and resources ( Khare *et al*., 2014). Here, we present a tool, the Abstract Sifter, built to improve efficiency in searching PubMed. Specifically, this tool was designed with the following objectives: 1) To make it quicker and easier to find relevant articles in PubMed; 2) To visualize the “literature landscape”, which can help focus on key relevant articles; 3) To make it easier to evaluate and take notes on abstracts; and 4) To facilitate collaboration on literature tasks.

## Methods

The Abstract Sifter application is a Microsoft Excel macro-enabled workbook that has been tested in Excel 2013 and 2016 on the Windows platform. Visual Basic for Applications (VBA) was used to develop the features that go beyond native Excel functionality. For the retrieval of PubMed query results, Entrez Programming Utilities (E-utilities) ( Sayers, 2016) are called from VBA. These utilities were developed by the National Center for Biotechnology Information (NCBI) to allow software developers to query PubMed and other NCBI databases and retrieve the results for incorporation into local applications (2017). Through implementation as an Excel workbook, the Abstract Sifter can easily be shared with collaborators.

## Use case

**Figure 1.**
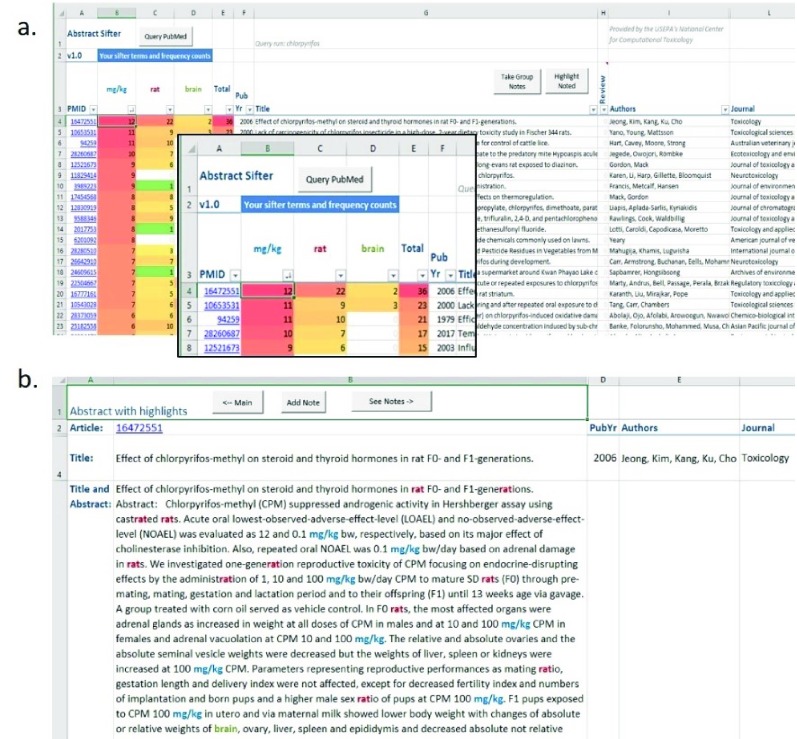
**1a.** Abstract Sifter Main sheet and **1b.** Abstract sheet view.

The Abstract Sifter application workbook contains seven sheets: ReadMe, Main, Abstract, Notes, Log, and Landscape, and SampleQueries. The Main sheet is where the basic functions operate, including the novel functionality called “sifting”. To start, the end-user clicks on the *Query PubMed* button at the top of the screen and enters any PubMed query of interest. For the example in Figure 1a, the end-user has entered the simple query “chlorpyrifos”. However, these queries can be more complex. In fact, any query run in PubMed can be executed in the Sifter. When the query entry is finished, the user then clicks on *Submit* and the query is sent to the NCBI PubMed E-utility. The first response returned by the E-utility is the number of articles found that satisfy the query. The citations are downloaded from PubMed by the Abstract Sifter, parsed by pattern matching algorithms coded in VBA. All citations are thus parsed for title, abstract, authors, publication year, journal, and PubMed identifier, and the data is inserted into rows in the Main sheet. Every new search will clear results from the previous query. For performance purposes, if the number of articles exceeds 5,000, the query will not be run and the user is encouraged to re-word the query to return fewer records.

The results of the query stored in the Main sheet can be browsed like any other data in a spreadsheet. The sifter feature provides a novel and effective way to narrow search results with large number of citations to find articles of interest. For example, the query for “chlorpyrifos” returned over 4,000 PubMed citations. If a researcher is looking for neurological effects in studies where rats were dosed with chlorpyrifos, the researcher could type the term “mg/kg” in the spreadsheet cell B3, “rat” in C3, and “brain” in D3 ( Figure 1a inset). The Abstract Sifter returns the number of occurrences of each term found in the title and abstract combined. The Main sheet’s citations can be sorted by these counts. Sifting by entering terms and sorting can be repeated. Similarly, new PubMed queries can be run, altered, and rerun. Double-clicking on any cell in the row (except the cell containing the PMID) takes the end-user to the Abstract sheet where the title and abstract of that citation are shown ( Figure 1b). The sifter terms are highlighted by giving each the color of the term on the Main sheet. Together, these query and sifting capabilities provide a powerful search tool.

**Figure 2.**
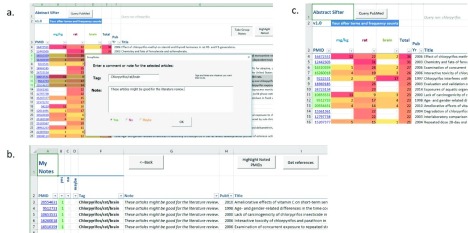
**2a.** Taking Group notes. **2b.** Viewing Notes sheet. **2c.** Highlighting noted citations.

The Abstract Sifter also incorporates functionality to allow the end-user to take notes on citations. On the Abstract sheet, for instance, the user can click on the button *Add Note*. A form appears that provides the opportunity to add short notes (tags) or long notes or to specify one of three categories (yes, no, or maybe) ( Figure 2a). How these values are used is a decision of the end-user. When the user clicks on *OK*, a row is inserted into the Notes sheet with the citation information along with the notes. Alternatively, the end-user can take notes on more than one article at a time from the Main sheet. To do this, the end-user selects multiple rows of interest and then clicks on *Take Group Notes*. Each of the selected citations will be inserted into the Notes sheet with the entered notes and tags ( Figure 2b).

Often, after entering a number of notes, the user will forget which citations have been read and evaluated and for which notes have been taken. By clicking on the *Highlight Noted PMIDs* button either on the Main or Notes sheet, the PubMed identifier (PMID) on the Main sheet will be set to the color specified in the Note form ( Figure 2c). Using the built-in Excel filtering feature, the color can be selected or sorted on to view Noted citations.

The Notes sheet itself can be viewed and edited and rows can be deleted. Entries on the Notes sheet can be exported to PubMed where they can then be downloaded in a number of different formats, including a format for direct import into citation management software. The button to export to a citation manager via PubMed is labelled *Get references* and appears on the Notes sheet.

**Figure 3.**
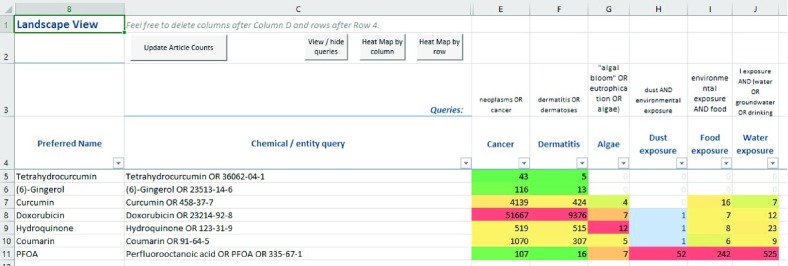
Landscape sheet.

Another unique feature of the Abstract Sifter is the Landscape sheet functionality. The Landscape sheet is an alternative to the Main sheet as an entry point, and provides the end-user a visualization of literature for a set of chemicals. To use this functionality, the end-user enters chemical name queries in Column C of the Landscape sheet after Row 4. An example in which the end-user has entered seven chemical queries is shown in Figure 3. The chemical queries can be extended with CAS registry numbers or synonyms. Next, the end-user enters subject matter query text in Row 3, Columns E and higher. In the example depicted in Figure 3, the end-user has entered several subject matter queries, starting with “neoplasms OR cancer”.

The end-user can then select cells in the intersection region (E5 through J11 in Figure 3) and click on the button *Update Article Counts*. This action causes the tool to iterate through the cells, build a query using the chemical terms in column C appended to the effect terms in Row 3, and then send the query to PubMed for execution. The counts of the articles satisfying each query are returned from PubMed and are inserted into the corresponding cells. To see the PubMed records, the user double-clicks on a cell in the intersection region. This action starts the PubMed query process and sends the results to the Main page for sifting. More chemicals, entries and/or and additional queries can be added by the user on this Landscape sheet. Buttons are available on the Landscape sheet to help with formatting results, such as applying heat-map coloring to the article counts. The SampleQueries sheet has some text that can be used as a starting point for Landscape queries. To use, the end-user selects rows and clicks on the *Send Queries to Landscape* button to have the queries appended to Row 3 on the Landscape page.

The Log sheet contains a row for each query run. The query text is inserted into the row along with date and time information and the number of records returned. Queries can be easily rerun by double-clicking on the query text in column C.

## Discussion

The Abstract Sifter can facilitate many PubMed literature tasks by enabling rapid identification, triage, and tracking of relevant articles. The literature landscape viewing and navigating capabilities give researchers distinctive insight into characteristics of a literature corpus.

## Software availability

The Abstract Sifter Excel workbook and user documentation are available at: https://github.com/USEPA/CompTox-Chemistry-Dashboard-Abstract-Sifter/

Archived source code as at the time of publication: http://dx.doi.org/10.5281/zenodo.1040961 ( nancycolebaker, 2017)

License: CC0 1.0 Public Domain Dedication.

## References

[ref-2] Khare R Leaman R Lu Z: Accessing biomedical literature in the current information landscape. *Methods Mol Biol.* 2014;1159:11–31. 10.1007/978-1-4939-0709-0_2 24788259 PMC4593617

[ref-4] nancycolebaker: nancycolebaker/LitInfo: PubMed Abstract Sifter public release v.1.(Version v1). *Zenodo.* 2017 Data Source

[ref-1] NCBI Resource Coordinators: Database Resources of the National Center for Biotechnology Information. *Nucleic Acids Res.* 2017;45(D1):D12–D17. 10.1093/nar/gkw1071 27899561 PMC5210554

[ref-3] Sayers E: A General Introduction to the E-utilities. 2016; Retrieved 2016. Reference Source

