# Supplementary material for: Abstract Sifter: a comprehensive front-end system to PubMed
Source: F1000Res. 2017 Dec 21;6:Chem Inf Sci-2164. [Version 1] doi: 10.12688/f1000research.12865.1 (PMC5801564; doi:10.12688/f1000research.12865.1)
Supplement: Peer review history

# Peer review: Referee response for version 1

**DOI:** 10.5256/f1000research.13943.r30158

**Published:** 2018-02-06

**Authors:** Qingliang (Leon) Li

**Affiliations:** 1 National Institutes of Health, Bethesda, MD, USA

The authors reported an Excel front-end for PubMed search by using NCBI E-Utilities. The tool supports searching literature in PubMed, importing search results into excel, adding notes into individual literature etc. Here are some minor issues that the authors may consider to improve the usabilities of the tool: 

1. Currently, the tool has a limit of 5000 results for a search. This makes sense when considering excel performance, however, it will improve the usability if the tool can support pagination and/or have the user to select which literature to import.
2. The term search/count feature in the main sheet can be improved by restricting the "searched term" to be a "real term".  For example, "gene **rat**ion" that has nothing related to "rat" should not be highlighted as a 'rat term'. 
3. In the landscape view, the authors may consider linking chemical name to chemical databases, such as PubChem, to view chemical structures. 
4. The authors may consider connecting this tool with "My NCBI" ( https://www.ncbi.nlm.nih.gov/account/?back_url=https%3A%2F%2Fwww.ncbi.nlm.nih.gov%2Fmyncbi%2F)

I have read this submission. I believe that I have an appropriate level of expertise to confirm that it is of an acceptable scientific standard.

# Peer review: Referee response for version 1

**DOI:** 10.5256/f1000research.13943.r29810

**Published:** 2018-01-15

**Authors:** Pauliina Damdimopoulou, Astrud Tuck

**Affiliations:** 1 Unit of Toxicological Sciences, Swetox, Karolinska Institutet, Södertälje, Sweden

This article describes a Microsoft Excel workbook based tool for rapid PubMed literature searches where the results can be easily sifted and annotated. With the ever-growing body of literature, tools for finding and sorting literature become increasingly more important. I am not a software developer myself, and cannot therefore evaluate the technical aspects of the tool (codes, macros, algorithms…). However, I am a keen end-user of tools such as Abstract Sifter, because my work is highly interdisciplinary and I often need to rapidly find information about unfamiliar topics, and can therefore estimate the user friendliness and usefulness of this new tool.

 A clear benefit of Abstract Sifter is its Excel format. Most researchers are familiar with Excel and therefore starting to work with Abstract Sifter does not require any extra effort to understand the interphase. It is convenient that different searches can be saved and shared with colleagues like any file. I can also vision sharing a Sifter file through a cloud service and simultaneously annotating and going through literature for example for a review or grant application.

 I have carefully read through the article (last updated 02 JAN 2018) and the User Guide (version 1.0) as well as tried the different functions of Abstract Sifter (v1), and find only minor issues to comment on. I think the work is of acceptable standard to be approved.

 Minor comments

1. Building queries in the Sifter. It would be helpful to have an ”advanced query builder” option available. When I was trying different queries, I ended up building the more complicated ones using the PubMed advanced search builder and copy pasting the query line to Sifter.
2. Does the Sifter function take article keywords and authors into consideration in the counts?
3. Page 3: ”Highlight Noted PMIDs” should be ”Highlight Noted”.
4. The ”For HAWC” button on the Notes sheet is not explained in the article (please also see comment 7).
5. The Helpful Tips in the user guide are very useful! One to add could be how to retrieve selected references from PubMed with the ”Get references” button. For example, if I have gone through and annotated the 4,000 chlorpyrifos citations and only want to retrieve the 150 I’ve marked ”yes” (green) in the Notes sheet, how should I do that?
6. The Landscape function is an exciting way to quickly get an idea of how well certain topics have been covered in the literature. What is Column D ”Link” in the Landscape sheet? What is the relationship between Rows 3 and 4? Is row 4 just user defined short names for row 3 queries, and not taken into account in the PubMed based search for citations? This could be clarified in the article text. The Landscape heatmap colours could also be explained too together with their cut-offs (I assume it’s based on % instead of absolute counts?). Should 0 labelled with white or blue?
7. On the ReadMe sheet in the Shifter file, ”Chemical sheet” is mentioned, although the Sifter does not have one with that name. Do you maybe mean Landscape sheet? ReadMe page also gives a link to a HAWC video, although HAWC is not explained in the article (see also comment 4).
8. User Manual page 10: ”…short version of the chemical name is in Column A” should be ”…Column B”. Column A is called ”DSSTOX link to Dashboard” in the file.
9. I wonder, if I were to use this tool with a colleague to select citations for a review, for example, is there a way to add ”comments” to the notes? For example, if I have classified a citation as ”yes” but my colleague does not agree, how could that be easily marked into the Notes page?
10. Does the Shifter work both in Mac and PC environment? What about Open Office?

We have read this submission. We believe that we have an appropriate level of expertise to confirm that it is of an acceptable scientific standard.
